# A Parameter Sensitivity Analysis on Multiple Finite Element Knee Joint Models

**DOI:** 10.3389/fbioe.2022.841882

**Published:** 2022-05-26

**Authors:** Nynke B. Rooks, Thor F. Besier, Marco T. Y. Schneider

**Affiliations:** ^1^ Auckland Bioengineering Institute, University of Auckland, Auckland, New Zealand; ^2^ Department of Engineering Science, Faculty of Engineering, University of Auckland, Auckland, New Zealand

**Keywords:** sensitivity analysis, knee modeling, finite element modeling, contact mechanics, tibiofemoral joint

## Abstract

The reproducibility of computational knee joint modeling is questionable, with models varying depending on the modeling team. The influence of model variations on simulation outcomes should be investigated, since knowing the sensitivity of the model outcomes to model parameters could help determine which parameters to calibrate and which parameters could potentially be standardized, improving model reproducibility. Previous sensitivity analyses on finite element knee joint models have typically used one model, with a few parameters and ligaments represented as line segments. In this study, a parameter sensitivity analysis was performed using multiple finite element knee joint models with continuum ligament representations. Four previously developed and calibrated models of the tibiofemoral joint were used. Parameters of the ligament and meniscus material models, the cartilage contact formulation, the simulation control and the rigid cylindrical joints were studied. Varus-valgus simulations were performed, changing one parameter at a time. The sensitivity on model convergence, valgus kinematics, articulating cartilage contact pressure and contact pressure location were investigated. A scoring system was defined to categorize the parameters as having a “large,” “medium” or “small” influence on model output. Model outcomes were sensitive to the ligament prestretch factor, Young’s modulus and attachment condition parameters. Changes in the meniscus horn stiffness had a “small” influence. Of the cartilage contact parameters, the penalty factor and Augmented Lagrangian setting had a “large” influence on the cartilage contact pressure. In the rigid cylindrical joint, the largest influence on the outcome parameters was found by the moment penalty parameter, which caused convergence issues. The force penalty and gap tolerance had a “small” influence at most. For the majority of parameters, the sensitivity was model-dependent. For example, only two models showed convergence issues when changing the Quasi-Newton update method. Due to the sensitivity of the model parameters being model-specific, the sensitivity of the parameters found in one model cannot be assumed to be the same in other models. The sensitivity of the model outcomes to ligament material properties confirms that calibration of these parameters is critical and using literature values may not be appropriate.

## 1 Introduction

Computational knee joint models can give valuable insight into the mechanics of the knee joint and have increased in popularity over the last few decades (Erdemir et al., 2019). However, developing these models involves a complex process where many decisions have to be made, which makes the reproducibility of these models questionable. In a previous study, five modeling teams were given the same datasets to develop a computational knee joint model simulating 0–90 degrees of flexion (Rooks et al., 2021). Decisions made by the teams, such as interpretation of data and workflow preferences, resulted in variances in the models, including the representations of the knee joint structures, the material models, and the anatomical coordinate systems used. To understand the influence that these variances have on the validity and reproducibility of the models, it is important to conduct a sensitivity analysis of the model parameters on the model outcomes. This analysis can provide valuable insight into which model parameters should be model-specific or subject-specific and which parameters could be adequately captured with a generic, or population-based value. This has important implications for model reproducibility and the standardization of model development and calibration processes.

Sensitivity analyses on finite element models of the knee joint were previously performed, focusing on different model aspects, including material model properties (e.g., Gu and Pandy, 2020), ligament attachment sites (e.g., Beillas et al., 2007), geometries (e.g., Li et al., 2001), and parameters with no physical meaning (Bernakiewicz and Viceconti, 2002). Often the effects of these parameters on kinematics, contact mechanics, or tissue responses (e.g., stresses) were studied. Some sensitivity analyses used multiple models (Bloemker et al., 2012; Gu and Pandy, 2020), but most publications were based on one model, where the results might not be translatable to other models. Most sensitivity analyses in the literature used models with springs or line elements to represent the ligaments, with only a few using continuum representations of the ligaments (Song et al., 2004; Dhaher et al., 2010).

This study presents a parameter sensitivity analysis on multiple finite element knee joint models with continuum ligament representations to explore the importance of parameter sensitivity in patient-specific modeling of the knee joint. Parameters of the ligament and menisci, tibiofemoral cartilage contact formulation, rigid cylindrical joint (RCJ), and simulation control settings were studied to obtain a broad understanding of model sensitivity.

## 2 Methods

### 2.1 Models Used

**FIGURE 1:**
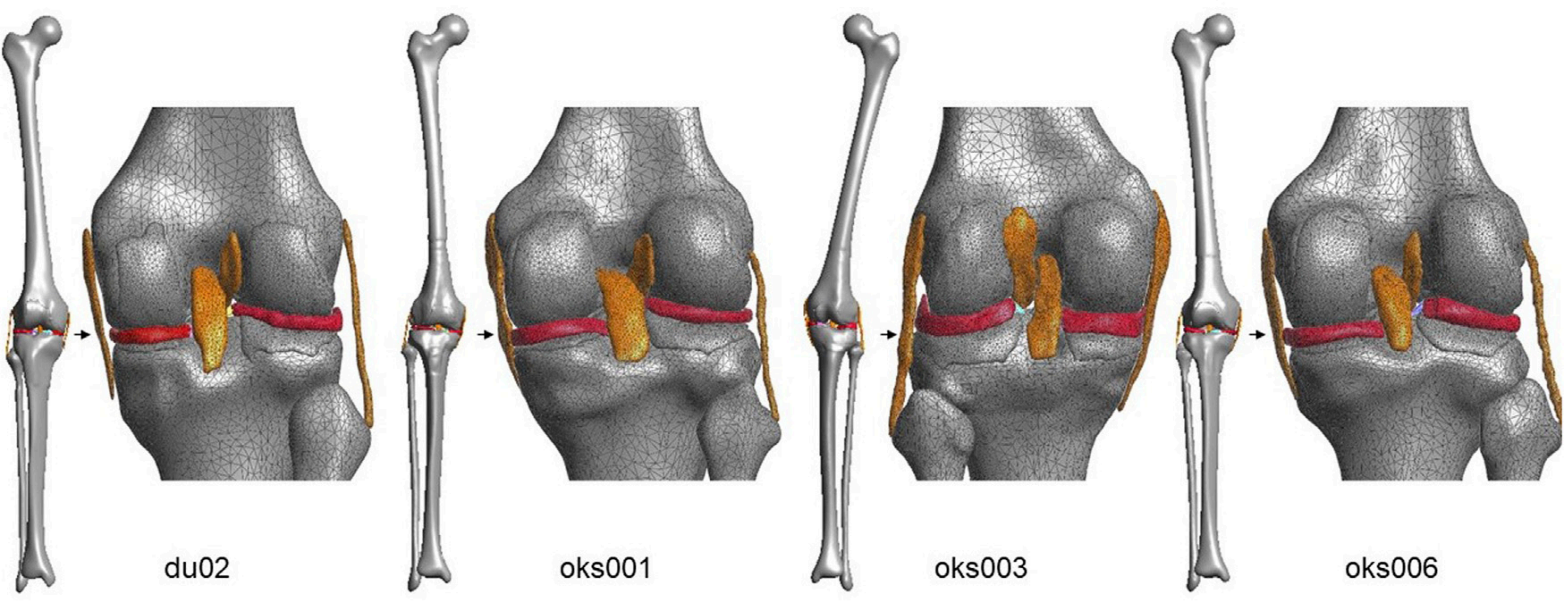
Anterior (full-length bones) and posterior (articulating region) view of the four models.

Four tibiofemoral joint finite element models were used (Table 1 and Figure 1), which were previously developed and calibrated using cadaveric MRI and laxity datasets obtained from the Open Knee(s) project (specimens oks001, oks003 and oks006) (Bennetts et al., 2015; Bonner et al., 2015; Colbrunn et al., 2015; Erdemir et al., 2015; https://simtk.org/projects/openknee) and the Natural Knee Data repository (specimen du02) (Harris et al., 2016; Ali et al., 2016; https://digitalcommons.du.edu/natural_knee_data/). The full description of the model development and calibration workflow is available on https://simtk.org/projects/abi_knee_models (Documents section), and is summarized here.

**TABLE 1:** Subject data characteristics.

Model Number	Sex	Age (yrs)	Height (m)	Mass (kg)
du02	Male	44	1.83	70.31
oks001	Male	71	1.83	77.10
oks003	Female	25	1.73	68
oks006	Female	71	1.524	49.4

The segmentations of the bones, cartilages, ligaments and menisci were obtained from the Open Knee(s) dataset for the Open Knee(s) specimens, and segmented from MRI data for the Natural Knee Data specimen. Full-length bone meshes of the femur, tibia, and fibula were initially predicted using statistical shape models (Zhang et al., 2016a; Zhang et al., 2016b). These bone meshes were registered to and combined with the segmented data, then remeshed to triangulated surface elements. The cartilage, ligament and meniscus meshes (tetrahedral elements) were generated in FEBioStudio (version 1.0.0, Maas et al., 2012). The model was assembled and run in FEBio (version 3.0.0, Maas et al., 2012) with sliding-elastic contacts between the articulating cartilages and menisci. The ligament meshes were rigidly tied to the bone meshes. An anatomical coordinate system based on the Grood & Suntay description was used (Grood and Suntay, 1983). RCJs were embedded to prescribe loads or displacements in the six degrees of freedom (DOF) of the knee joint coordinate system. The bones and cartilages were modeled as rigid bodies, the menisci as Fung-orthotropic materials and the ligaments as Neo-Hookean materials. To calibrate the model, the ligament prestretch factors and the ligament Young’s moduli were optimized to resemble the cadaveric laxity data in an anterior-posterior, internal-external and varus-valgus simulation with the knee joint in full extension.

### 2.2 Simulations Performed

For each model parameter changed, a simulation was run, bringing the tibiofemoral joint into contact, applying 0.5 body weight of axial load, then applying varus-valgus moments of 40 Nm (Table 2). Simulations were run on High-Performance Computers (Intel(R) Xeon(R) Gold 6136 CPU @ 3.00 GHz 1024 GB Ram).

**TABLE 2:** Simulations performed.

	Simulation performed	Time steps
1	Prestretch application	0.0–0.1
	*oks models*: Rotate to robot data flexion angle	0.1–0.5
	*oks models*: Apply -20N axial load	0.5–1.0
	*du02 model*: Rotate to robot data flexion angle	0.1–1.0
2	Rotation to 0 degrees of knee flexion	1.0–1.5
3	Application of 0.5 body weight axial load (model specific)	1.5–2.0
4.1	Valgus moment applied: 40,000 Nmm	2.0–3.0
4.2	Varus moment applied: −40,000 Nmm	2.0–3.0

### 2.3 Model Parameters of Interest

The parameters of interest included ligament and meniscus material properties, parameters of the sliding elastic tibiofemoral cartilage contact, simulation control parameters, and parameters of the RCJs (Table 3). One parameter of interest was changed at a time. In total, 101 varus and valgus simulations were performed per model.

**TABLE 3:** Parameters of interest and their values investigated. Ligament abbreviations: anterior and posterior cruciate ligaments (ACL & PCL) and medial and lateral collateral ligaments (MCL & LCL).

Category	Parameter of interest	Original	Investigated range	Step size	Investigated values	Number of simulations per model
Ligament and meniscus material properties	Ligament prestretch factor ACL, PCL, MCL and LCL	Model-specific calibrated value	Calibrated value ± 0.1	0.025		32
	Ligament Young’s modulus ACL, PCL, MCL and LCL	Model-specific calibrated value	Calibrated value ± 100 MPa	25		32
	Ligament attachment condition ACL, PCL, MCL and LCL	Rigid tied contact node sets number 1	2 node sets (1 = original & 2 = half of the original nodes) on the superior and inferior ligament attachment site			12
	Meniscus horn stiffness (18 springs per horn attachment)	1 N/mm each spring	1–20 N/mm each spring		5, 10 & 20 N/mm each spring	3
Tibiofemoral cartilage contact formulation (Sliding-elastic in FEBio)	Augmented Lagrangian	0 (Penalty method)			1 (Augmented Lagrangian)	1
	Penalty factor	1			0.5, 2, 5, 10	4
	Auto penalty	0 (Disabled)			1 (Enabled) with penalty factor = 1	1
	Two-pass	1 (Enabled)			0 (Disabled)	1
	Search radius	0.005			1	1
Simulation control parameters	Quasi-Newton update method	Full Newton			BFGS & Broyden with max_ups = 10	2
	Displacement tolerance	0.01			0.001	1
Rigid cylindrical joint (RCJ)	Force penalty (stiffness)	10,000 N/mm			5,000, 20,000 N/mm	2
	Moment penalty (torsional stiffness)	3,000,000 Nmm/radians			5,000, 10,000 & 20,000 Nmm/radians	3
	Gap tolerance	0.01			0.0001, 0.001 & 0.1	3
	Angular tolerance	0.0001			0.001, 0.01 & 0.1	3

#### 2.3.1 Ligament Attachment Condition

**FIGURE 2:**
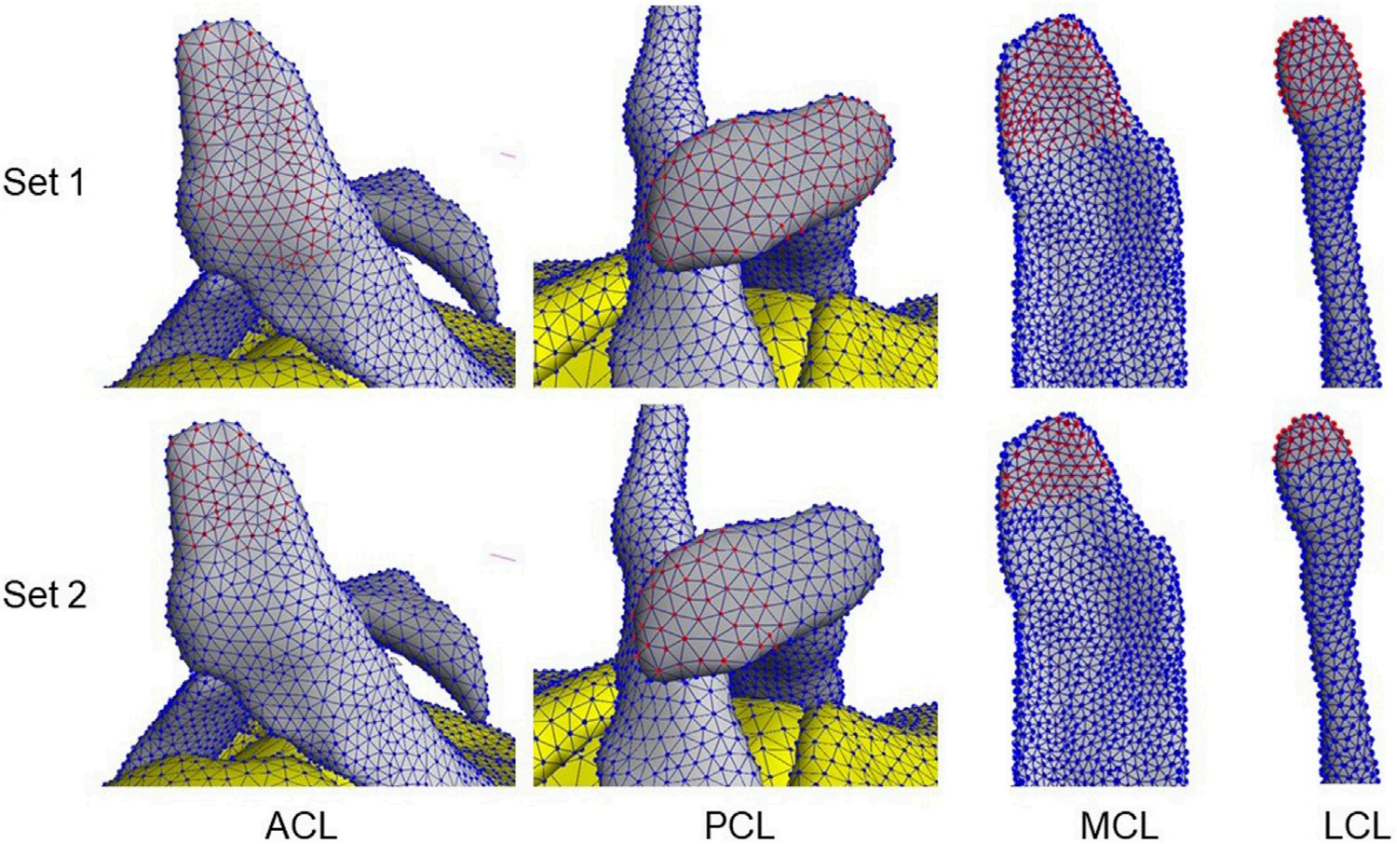
Examples of ligament attachment node sets 1 & 2 involved in the ligament–bone tied contacts. Red nodes indicate selected nodes in the set.

The anterior and posterior cruciate ligaments (ACL & PCL) and the medial and lateral collateral ligaments (MCL & LCL) were studied. To change the ligament attachment condition, the original number of nodes involved in the superior and inferior ligament-bone rigid tied contact (Set 1, Table 4) was halved (Set 2), as shown in Figure 2. For Set 2, the most proximal half of the original nodes in the ligament-femur rigid tied contact, and the most distal half of the original nodes in the ligament-tibia/fibula rigid tied contact was selected. For each ligament, three simulations were performed: femur ligament attachment Set 1 with tibia/fibula attachment Set 2, femur ligament attachment Set 2 with tibia/fibula attachment Set 2, and femur ligament attachment Set 2 with tibia/fibula attachment Set 1.

**TABLE 4:** The number of nodes in the original ligament attachment rigid tied contact (Set 1) per model. The number of nodes in Set 2 is half the number of nodes in Set 1.

	ACL-tib	ACL-fem	PCL-tib	PCL-fem	MCL-tib	MCL-fem	LCL-fib	LCL-fem
du02	58	173	55	64	122	70	38	58
oks001	52	124	61	98	114	159	27	23
oks003	42	161	42	64	170	105	31	51
oks006	39	46	48	71	196	74	27	20

### 2.4 Analysis

#### 2.4.1 Outcome Parameters

To analyze the sensitivity of the models to the parameters of interest, the model convergence, valgus kinematics, peak contact pressure, and location of peak contact pressure were studied. The six DOF kinematics during the varus and valgus simulations were calculated, but only the varus-valgus kinematics were used for analysis.

The peak contact pressure was calculated at a time step to which all simulations converged. This time step differed per parameter of interest and had to be larger than 2.25 (corresponding to 10,000 Nmm varus or valgus moment applied). If a simulation did not converge past a timestep of 2.25, this simulation was excluded from the analysis. The contact pressures were analyzed on the articulating surfaces of the tibial cartilages, and the medial and lateral tibial cartilages were analyzed separately.

To obtain the peak contact pressure, the faces in the tibial cartilage articulating surface with a contact pressure larger than 0 N/mm were selected and ordered from lowest to highest contact pressure. The average of the top 10% of the faces with the highest contact pressure was calculated as the peak contact pressure. The location of peak contact pressure was calculated by taking the average location of the faces included in the top 10%, to avoid any unusually high stresses from single deformed elements (Pal et al., 2019).

#### 2.4.2 Comparisons

**FIGURE 3:**
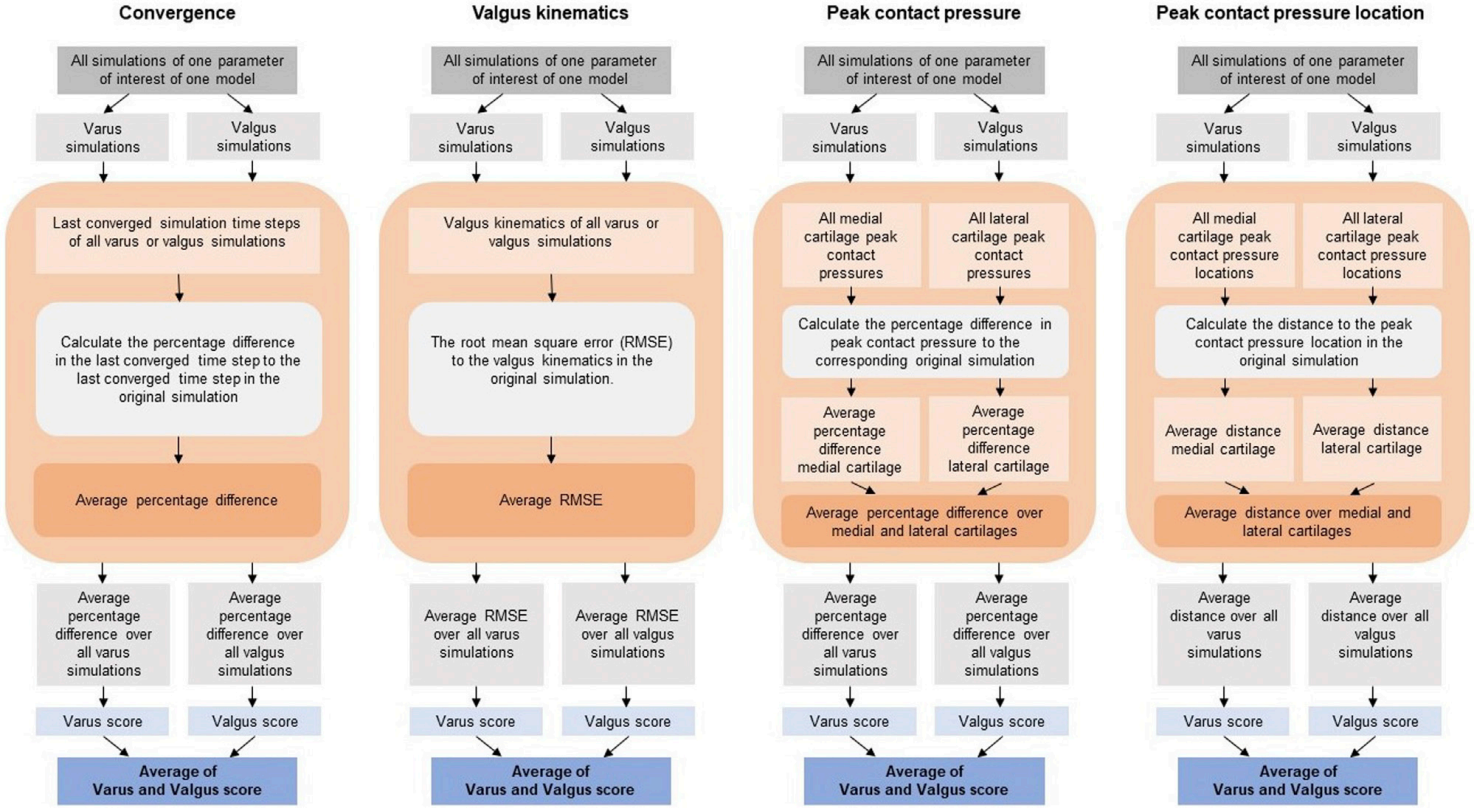
Analysis workflows of the four outcome parameters.

The influence of changing the parameters of interest on the outcome parameters was analyzed and compared to the original simulations (Figure 3). To quantify the convergence outcome parameter, the percentage difference in the last converged time step in the simulations of interest compared to the last converged time step in the original simulation was calculated. The average percentage difference of the simulations of the parameter of interest was calculated for the varus and valgus simulations separately.

The root mean square error (RMSE) between the valgus kinematics in the original simulation and the simulation of interest was calculated. The RMSE was calculated over all converged time steps of the simulation of interest. Only the time steps for which both the simulation of interest and the original simulation converged were taken into account. Since the time steps at which the model converged can be different between the original and the simulation of interest, the valgus kinematics of the original simulation were interpolated between converged time steps. The average RMSE of the simulations of the parameter of interest was calculated for the varus and valgus simulations separately.

The percentage difference in contact pressure of the simulation of interest compared to the original simulation was calculated. The percentage was calculated for the medial and lateral tibial cartilage separately, after which the average was obtained. The average percentage difference of the simulations of the parameter of interest was calculated for the varus and valgus simulations separately.

The peak contact pressure location was quantified by calculating the distance between the peak contact pressure location of the simulation of interest and the original simulation. The distances were calculated for the medial and lateral tibial cartilages separately, after which the average was obtained. The average distance of the simulations of the parameter of interest was calculated for the varus and valgus simulations separately.

##### 2.4.2.1 Qualitative Comparisons

To compare between the four outcome parameters, the quantitative results were scored on a scale from 0 to 3 (Table 5). The average quantitative scores of the varus and valgus simulations were rated separately, and their average score of each parameter of interest was analyzed. The four models were rated separately.

**TABLE 5:** Rating scores used to qualitatively rate the influence of the parameters of interest on the four model outcome parameters.

Influence score	Average percentage difference between last converged time steps (%)	Average valgus kinematics RMSE (deg.)	Average percentage difference between peak contact pressures (%)	Average distance between peak contact pressure locations (mm)
0: None	x = 0	x = 0	x = 0	x = 0
0.5: Negligible	0 < *x* ≤ 1	0 < *x* ≤ 0.2	0 < *x* ≤ 1	0 < *x* ≤ 0.2
1: Small	1 < *x* ≤ 5	0.2 < *x* ≤ 0.5	1 < *x* ≤ 5	0.2 < *x* ≤ 0.5
1.5: Small—Medium	5 < *x* ≤ 10	0.5 < *x* ≤ 0.9	5 < *x* ≤ 10	0.5 < *x* ≤ 1.25
2: Medium	10 < *x* ≤ 20	0.9 < *x* ≤ 1.4	10 < *x* ≤ 20	1.25 < *x* ≤ 2.5
2.5: Medium—Large	20 < *x* ≤ 40	1.4 < *x* ≤ 2	20 < *x* ≤ 40	2.5 < *x* ≤ 4
3: Large	x > 40	x > 2	x > 40	x > 4

## 3 Results

**FIGURE 4:**
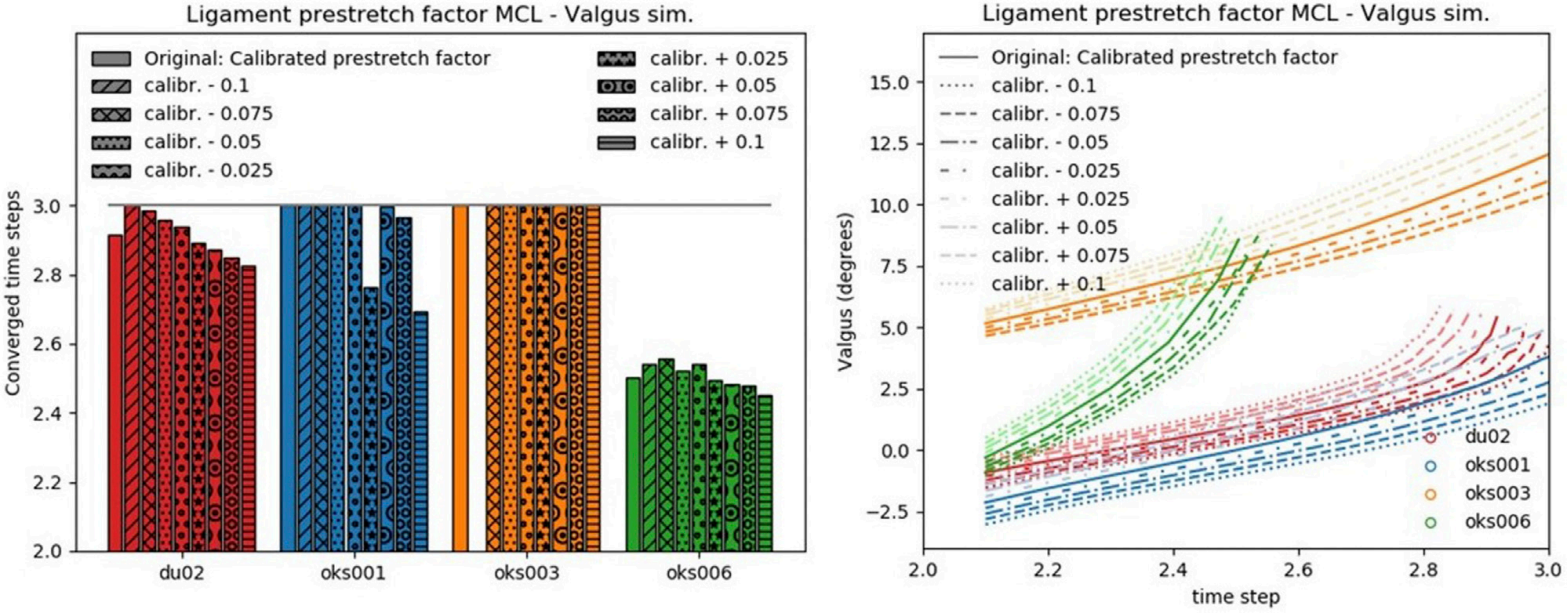
Convergence (left) and valgus kinematics (right) results for all valgus simulations of the Ligament prestretch factor MCL parameter.

**FIGURE 5:**
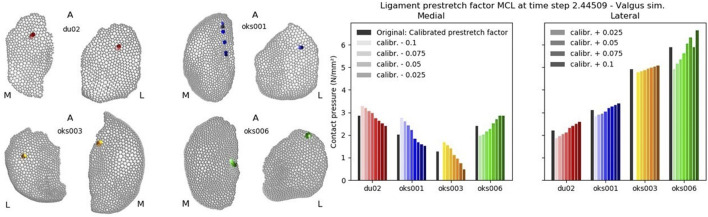
Peak contact pressure location (left) and peak contact pressure (right) results for all valgus simulations of the Ligament prestretch factor MCL parameter.

For each parameter of interest, all simulations’ convergence, valgus kinematics, peak contact pressures, and peak contact pressure location results were plotted. Examples of these plots are shown in Figures 4, 5. The plots of all simulations and analysis results can be found on https://simtk.org/projects/abi_knee_models (Documents section).

### 3.1 Ligament and Meniscus Material Properties

#### 3.1.1 Ligament Prestretch Factors

**FIGURE 6:**
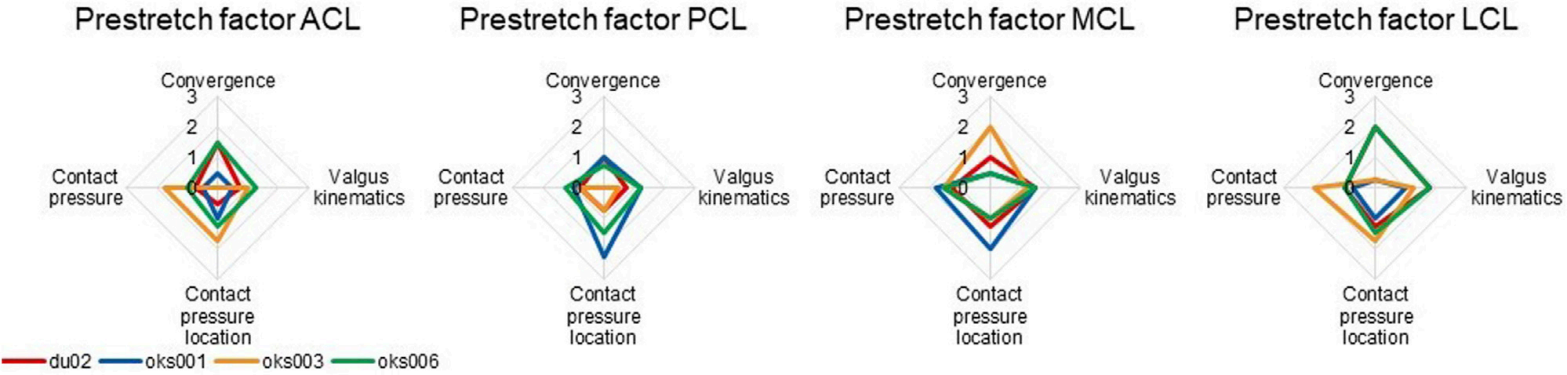
Ligament prestretch factor sensitivity analysis simulation results rated on influence on convergence, valgus kinematics, peak contact pressure, and location of peak contact pressure (0 (No influence) to 3 (Large influence)).

The influence of the ligament prestretch factor on the four outcome parameters was different between the four ligaments (Figure 6). In general, the four models showed different sensitivities to the same ligament prestretch factor on all four outcome parameters, but the influence of the MCL prestretch factor on contact pressure and valgus kinematics was similar across all models. The LCL prestretch factor had a larger influence on valgus kinematics in the varus (rated 1.5 to 2) compared to the valgus simulations (rated 0.5 to 1.5), whereas the MCL prestretch factor had a larger influence on valgus kinematics in the valgus simulation (rated 1.5 to 2) compared to the varus simulations (rated 0.5 to 1.5).

#### 3.1.2 Ligament Young’s Modulus

**FIGURE 7:**
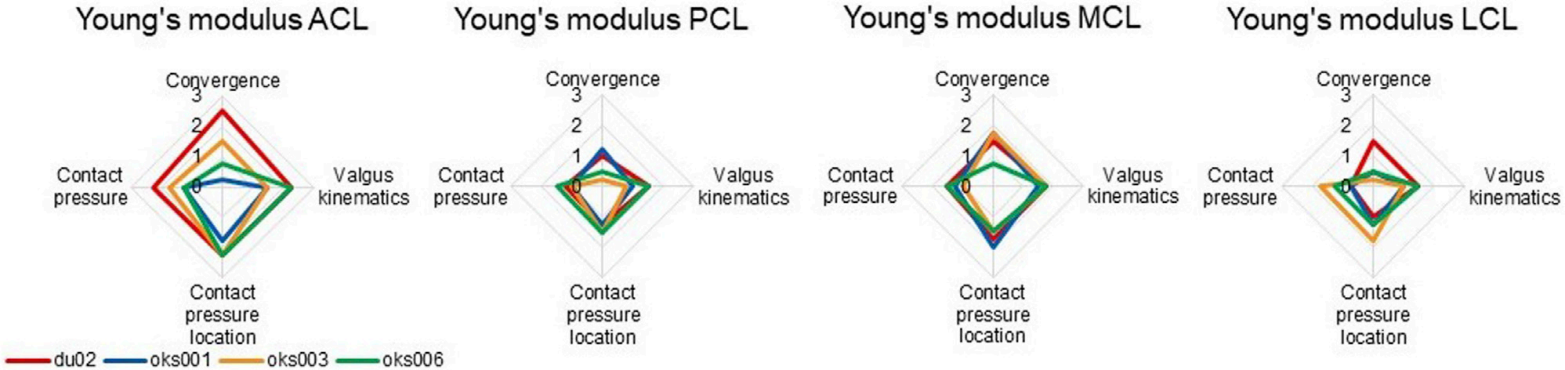
Ligament Young’s modulus sensitivity analysis simulation results rated on influence on convergence, valgus kinematics, peak contact pressure, and location of peak contact pressure (0 (No influence) to 3 (Large influence)).

Differences in sensitivities to ligament Young’s modulus on outcome parameters were found between the four models (Figure 7), for example, in the sensitivity on the contact pressure location. The exception was the sensitivity to the Young’s modulus of the MCL, which was consistent over all four models, except on convergence. The Young’s moduli of all ligaments had up to a medium influence on contact pressure for all models, except for the ACL Young’s modulus of model du02, which showed a medium to large influence.

#### 3.1.3 Ligament Attachment Condition

**FIGURE 8:**
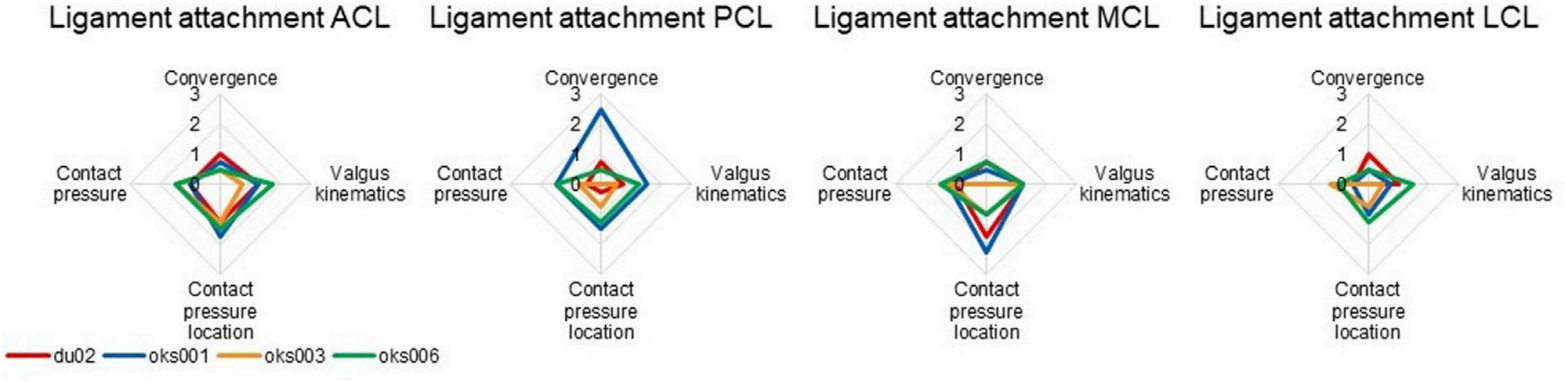
Ligament attachment condition sensitivity analysis simulation results rated on influence on convergence, valgus kinematics, peak contact pressure, and location of peak contact pressure (0 (No influence) to 3 (Large influence)).

For all models and all ligaments, except for ligament attachment condition of the PCL and MCL of model oks001, the change in ligament attachment condition had up to a medium influence on convergence, valgus kinematics, contact pressure location, and contact pressure (Figure 8). The convergence of model oks001 was very sensitive to the ligament attachment condition of the PCL. The model did not converge far enough to start the varus and valgus simulations for two out of three ligament attachment condition combinations.

#### 3.1.4 Meniscus Horn Stiffness

The influence of the meniscus horn stiffness on convergence, valgus kinematics, contact pressure location, and peak contact pressure was small.

### 3.2 Tibiofemoral Cartilage Contact Formulation

**FIGURE 9:**
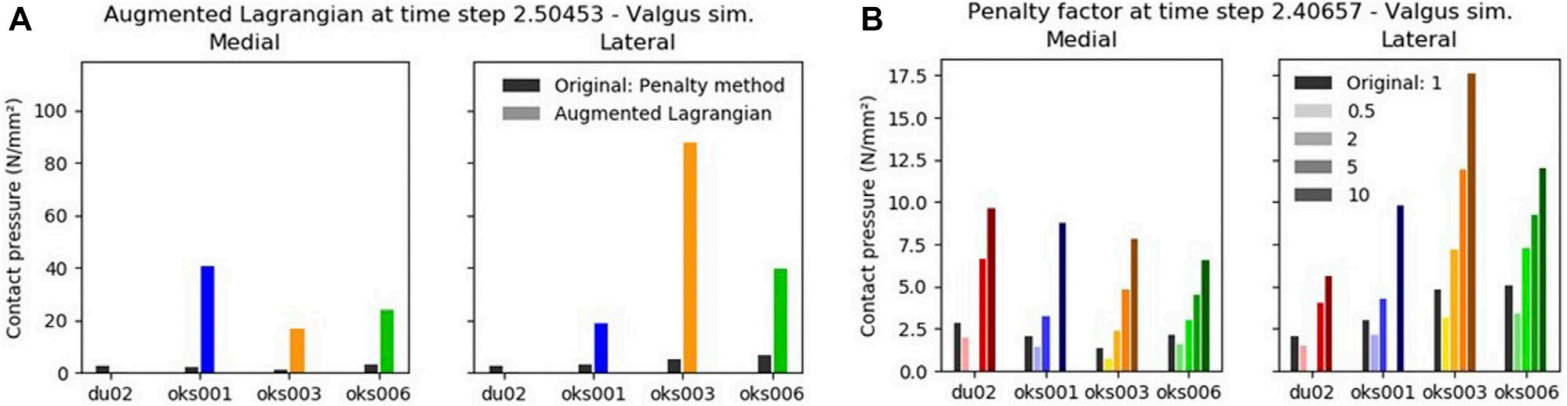
The influence of the Augmented Lagrangian **(A)** and Penalty factor **(B)** on the peak contact pressure in valgus rotation.

**FIGURE 10:**
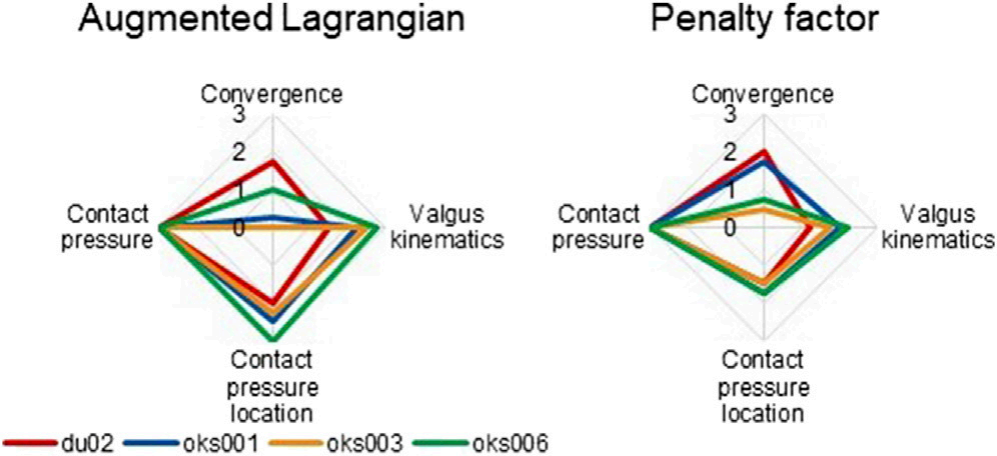
Tibiofemoral cartilage contact parameters sensitivity analysis simulation results rated on influence on convergence, valgus kinematics, peak contact pressure, and location of peak contact pressure (0 (No influence) to 3 (Large influence)).

With the Augmented Lagrangian contact formulation enabled, the area of contact pressure decreased, resulting in high contact pressure values (Figure 9A). For example, in the varus simulation of model oks003, the medial and lateral peak contact pressure values increased with an average of 2,454% compared to the original peak contact pressure. Therefore, the Augmented Lagrangian had a large influence on the peak contact pressure for all models (Figure 10). The Augmented Lagrangian had a medium to large influence on contact pressure location for all models. Model du02 did not converge in the valgus simulations (until time step 2.091). The penalty factor had a large influence on the contact pressure for all models (Figures 9B, 10).

For all models, the simulations exploring the sensitivity of the auto penalty and search radius parameters did not converge far enough to start the varus and valgus simulations. The simulations with the two-pass parameters disabled did converge and produced anatomically unrealistic outcomes such as cartilage penetration with no contact pressures present at the penetrating areas.

### 3.3 Simulation Control Parameters

Changing the Quasi-Newton update method to BFGS or Broyden instead of the original Full Newton resulted in models du02 and oks006 not converging, whereas the other two models converged with negligible changes to valgus kinematics, contact pressure location and peak contact pressure. Up to a small influence of changing the displacement tolerance on outcome parameters was found in all models, except for model oks001, for which no influence was found.

### 3.4 Rigid Cylindrical Joint Parameters

When changing the RCJ moment penalty, all models except for model oks003 failed to converge far enough to start the varus and valgus simulations. Not all of the simulations of model oks003 converged entirely either. However, when the model converged, up to a small influence on the other outcome parameters was found. The RCJ force penalty and gap tolerance had up to a small influence on the outcome parameters. RCJ angular tolerance had up to a small influence on convergence and valgus kinematics for all models. Larger influences were found on the peak contact pressure (rated 0.75 to 1.5) and contact pressure location (rated 1 to 2).

## 4 Discussion

In this study, the sensitivity of the convergence, valgus kinematics, peak contact pressure, and peak contact pressure location of four knee finite element models to various modeling parameters was studied to explore the importance of parameter sensitivity in patient-specific modeling of the knee joint. To summarize, the main findings from this study are:

• The parameters resulting in converging simulations with the largest influence on the model outcomes were the ligament prestretch factor, ligament Young’s modulus, and parameters of the articulating cartilage contact formulation.
• For the majority of parameters, the sensitivity was model-specific. Therefore, the sensitivity of the parameters found in one model cannot be assumed to be the same in other models.
• The sensitivity of the model outcomes to ligament material properties confirms that calibration of these parameters is critical and using literature values may not be appropriate.

Differences in sensitivity between the models were found in the majority of modeling parameters studied. This indicates that the sensitivity of modeling parameters is model-dependent. For some parameters the trend of the influence on model outcomes was similar between the models. Knee joint finite element model sensitivity analyses reported in the literature typically present data from one model. Since the sensitivity to modeling parameters was found to be model-dependent in the majority of parameters studied, the sensitivities of the parameters found in one model cannot be assumed to be the same in other models.

As expected, the parameters with the greatest influence on the valgus kinematics included the ligament material properties. Often, literature values are used for the ligament material properties, however, our results suggest a critical need to calibrate ligament properties, across a range of conditions using suitable, preferably specimen-specific, data. In light of these results, literature values should only be used as starting points for model calibration. An influence on the valgus kinematics was often accompanied by an influence on the peak contact pressure and its location. This was expected since a different orientation of the bones will influence the area of the cartilages in contact, and therewith the peak contact pressure. The parameters had a variable, model dependent, effect on convergence, reflecting the influence of the parameter on the stability of the model. The model-specific sensitivity to convergence could be due to differences in mesh topology, which is one of the few model-specific aspects, whereas most other settings were the same between the models. Typically, model convergence is not discussed in papers on finite element knee joint modeling, even though it is a major problem to overcome in model development.

### 4.1 Ligament and Meniscus Material Properties

Our findings confirm that the material properties of the ligaments are sensitive parameters that influence the model outcomes. This is in agreement with previous studies, where the sensitivity of the model ligament prestretch factors (also known as prestrain, zero load length, or preload) on the kinematics (Bloemker et al., 2012; Germain et al., 2016; Esrafilian et al., 2020) and joint contact force (Esrafilian et al., 2020) was found. Sensitivity of the ligament stiffness on joint kinematics (Germain et al., 2016) and peak contact stress (Li et al., 2001) has also been reported in the literature. These findings further reinforce the need for specimen-specific model calibration.

For most models and most ligaments, the ligament attachment condition had up to a medium influence on the model outcomes. A sensitivity analysis by Beillas et al. (2007) found that the ligament insertion locations influenced the knee joint kinematics. Beillas et al. (2007) represented ligaments as line segment springs, where they changed the spring insertion points. In our sensitivity analysis, the ligament attachment condition was changed by halving the number of nodes involved in the ligament attachment tied contacts without changing the location of the ligament itself. Changing the ligament location itself would likely have a larger influence on the model outcomes. However, the ligaments were not altered from their position based on MR imaging to maintain anatomical accuracy.

The meniscus horn stiffness had a small effect on the outcome parameters. In a study by Haut Donahue et al. (2003), changing the meniscus horn stiffness influenced the contact pressure distribution. However, they used a meniscus horn stiffness range of 50–30,000 N/mm, which is much larger than the range in our sensitivity analysis (18–360 N/mm). Increasing the meniscus horn stiffness range in our study could have resulted in a larger influence on the outcome parameters. However, we did not deem a stiffness of 30,000 N/mm physiological.

### 4.2 Tibiofemoral Cartilage Contact Formulation

The penalty factor of the cartilage contact formulation influenced the model outcomes, which was expected since the penalty factor provides control of the contact traction forces and therewith penetration distance of the contact surfaces [FEBio user manual v3.0 (Maas et al., 2012)]. Enabling the Augmented Lagrangian also influenced the model outcomes. The peak contact pressure values increased, with the highest increase in model oks003 with an average of 2,454%. Using the Augmented Lagrangian method, the penalty factor is used to scale the Lagrange multiplier increment. According to the FEBio manual, this penalty factor often has to be smaller when using the Augmented Lagrangian compared to the penalty method. The penalty factor was not changed between methods in this study, which could have caused the large influence on the outcome parameters.

Disabling the two-pass parameter resulted in large penetration distances. Swapping the primary and secondary surfaces could potentially improve the contact when the contact is only calculated from one surface to the other instead of both ways. Convergence issues arose when increasing the search radius and enabling the auto-penalty contact parameters. The search radius in the original models was much smaller (0.005) than the default FEBio value (1.0). The original search radius value was copied from the Open Knee(s) model (https://simtk.org/projects/openknee) and resulted in an acceptable contact. The search radius is a scaling factor of a dimensional cut-off value to select point to facet projections included in the contact calculations. Due to curvature in the meshes, multiple distances could be calculated per point [FEBio user manual v3.0 (Maas et al., 2012)]. With the search radius value set to 1, these multiple projections could be selected, potentially causing convergence issues. This chance is reduced when using a lower search radius. None of the models converged when the auto penalty parameter was selected. Careful selection of the penalty factor is required if the auto penalty factor feature in FEBio is used. Typically, the penalty factor is not reported in the literature. The influence of the penalty factor on model convergence with the auto penalty parameter was not studied.

### 4.3 Simulation Control Parameters

The change in the Quasi-Newton update method had a large influence on convergence for two models but had a negligible influence on the outcome parameters if the simulations converged. For the outcome parameters it does not matter which Quasi-Newton update method is chosen, as long as the models converge. The BFGS and Broyden methods are controlled by the maximum number of stiffness updates and reformations (max_ups and max_refs) parameters. Changing these values could have potentially improved convergence.

### 4.4 Rigid Cylindrical Joint Parameters

Of the RCJ parameters, the largest influence on the outcome parameters was found by the moment penalty parameter, which caused convergence issues. The RCJ moment penalty represents the torsional stiffness of the RCJ. The original parameter values were based on the RCJ values in the Open Knee(s) (https://simtk.org/projects/openknee) model and were a lot higher than the values investigated in the sensitivity analysis.

### 4.5 Limitations

The LCL prestretch factor had a larger influence on the valgus kinematics in the varus compared to the valgus simulations, whereas the effect was opposite for the MCL prestretch factor. This was due to the LCL often being slack in the valgus simulation and the MCL often being slack in the varus simulation. This finding shows that the influence of the ligament parameter studied is dependent on the simulations performed. Therefore, performing sensitivity analyses for simulations of multiple DOF of the knee joint would be encouraged, whereas in this study the scope of the analysis was limited to varus-valgus simulations.

The qualitative rating scores used in this study were established specifically for this sensitivity study. The score that is assigned to a certain outcome parameter range influences the qualitative results directly, and a different scoring system is likely to affect the results. However, this method provided a means to be able to compare between outcome parameters. The models converged at different time steps, which means different loads were applied at the analyzed point in the simulation, which could have influenced the results. For analysis, the average rating of the varus and valgus simulations was taken, which might have influenced the conclusions due to different influences of the parameter in the two simulations.

This study is also limited to the modeling parameters that were investigated. Many parameters were not included, which potentially influences the outcome parameters. For example, in a previous study, the geometry and material properties of cartilage and the menisci influenced the joint pressure, the contact area, and the compressive load (Gu and Pandy, 2020). Other modeling decisions, such as mesh refinement, are typically explored but were not included in this study for the sake of brevity.

The four models included in this study were obtained using the same model development and calibration workflows. Sensitivity analyses might be dependent on these workflows, for example, on the FE solver software used. In this sensitivity analysis, the contact formulation and the RCJ parameters are specific to FEBio. To investigate the sensitivity of modeling parameters on models developed in other FE solver software and obtained with different workflows, further sensitivity analyses should be performed.

## 5 Conclusion

In this study, we gained insight into the influence of multiple knee joint finite element modeling parameters on model outcomes. The sensitivity of the model parameters was found to be model-specific. This indicates that the sensitivity of the parameters found in one model cannot be assumed to be the same in other models. To best understand the important parameters on a patient-specific basis, we suggest a sensitivity analysis to be performed for each model. The sensitivity of the model outcomes to ligament material properties confirms that calibration of these parameters is critical and using literature values may not be appropriate. Knowing the sensitivity of the multiple parameters can help with deciding which model parameters should have a model-specific or subject-specific value and which can be adequately captured using a generic value which could potentially be standardized improving model reproducibility. It is important to understand the influence of the parameters on the model outcomes, even if these influences are small, since all influences add up and could affect the conclusions drawn from the model.

## Data Availability

The datasets presented in this study can be found in online repositories. The names of the repository/repositories and accession number(s) can be found below: https://simtk.org/projects/abi_knee_models.

## References

[B1] Ali A. A. Shalhoub S. S. Cyr A. J. Fitzpatrick C. K. Maletsky L. P. Rullkoetter P. J. (2016). Validation of Predicted Patellofemoral Mechanics in a Finite Element Model of the Healthy and Cruciate-Deficient Knee. J. biomechanics 49 (2), 302–309. 10.1016/j.jbiomech.2015.12.020 PMC4761469 26742720

[B2] Beillas P. Lee S. W. Tashman S. Yang K. H. (2007). Sensitivity of the Tibio-Femoral Response to Finite Element Modeling Parameters. Comput. methods biomechanics Biomed. Eng. 10 (3), 209–221. 10.1080/10255840701283988 17558649

[B3] Bennetts C. J. Chokhandre S. Donnola S. B. Flask C. A. Bonner T. F. Colbrunn R. W. (2015). “Open Knee(s): Magnetic Resonance Imaging for Specimen-specific Next Generation Knee Models,” in SB3C2015, Summer Biomechanics, Bioengineering and Biotransport Conference, Utah, USA, June 17-20, 2015.

[B4] Bernakiewicz M. Viceconti M. (2002). The Role of Parameter Identification in Finite Element Contact Analyses with Reference to Orthopaedic Biomechanics Applications. J. biomechanics 35 (1), 61–67. 10.1016/S0021-9290(01)00163-4 11747884

[B5] Bloemker K. H. Guess T. M. Maletsky L. Dodd K. (2012). Computational Knee Ligament Modeling Using Experimentally Determined Zero-Load Lengths. Tobej 6, 33–41. 10.2174/1874230001206010033 22523522 PMC3325586

[B6] Bonner T. F. Colbrunn R. W. Chokhandre S. Bennetts C. Erdemir A. (2015). “Open Knee(s): Comprehensive Tibiofemoral Joint Testing for Specimen-specific Next Generation Knee Models,” in SB3C2015, Summer Biomechanics, Bioengineering and Biotransport Conference, Utah, USA, June 17-20, 2015.

[B7] Colbrunn R. W. Bonner T. F. Chokhandre S. K. Bennetts C. J. Halloran J. Erdemir A. (2015). “Open Knee(s): Comprehensive Patellofemoral Joint Testing for Specimen-specific Next Generation Knee Models,” in ASB 2015, 39th Annual Meeting of the American Society of Biomechanics, Columbus, Ohio, USA, August 5-8, 2015.

[B8] Dhaher Y. Y. Kwon T.-H. Barry M. (2010). The Effect of Connective Tissue Material Uncertainties on Knee Joint Mechanics under Isolated Loading Conditions. J. biomechanics 43 (16), 3118–3125. 10.1016/j.jbiomech.2010.08.005 PMC3641768 20810114

[B9] Erdemir A. Bennetts C. Bonner T. Chokhandre S. K. Colbrunn R. W. (2015). “Open Knee(s): Founding Data for Next Generation Knee Models,” in BMES/FDA, Frontiers in Medical Devices Conference: Innovations in Modeling and Simulation, Washington, DC, USA, May 18-20, 2015.

[B10] Erdemir A. Besier T. F. Halloran J. P. Imhauser C. W. Laz P. J. Morrison T. M. (2019). Deciphering the “Art” in Modeling and Simulation of the Knee Joint: Overall Strategy. J. biomechanical Eng. 141 (7), 071002. 10.1115/1.4043346 PMC6611350 31166589

[B11] Esrafilian A. Stenroth L. Mononen M. E. Tanska P. Avela J. Korhonen R. K. (2020). EMG-assisted Muscle Force Driven Finite Element Model of the Knee Joint with Fibril-Reinforced Poroelastic Cartilages and Menisci. Sci. Rep. 10 (1), 1–16. 10.1038/s41598-020-59602-2 32080233 PMC7033219

[B12] Germain F. Rohan P. Y. Rochcongar G. Rouch P. Thoreux P. Pillet H. (2016). “Role of Ligaments in the Knee Joint Kinematic Behavior: Development and Validation of a Finite Element Model,” in Computational Biomechanics for Medicine. Editors Joldes G. Doyle B. Wittek A. Nielsen P. Miller K. (Cham: Springer), 15–26. 10.1007/978-3-319-28329-6_2

[B13] Grood E. S. Suntay W. J. (1983). A Joint Coordinate System for the Clinical Description of Three-Dimensional Motions: Application to the Knee. J. biomechanical Eng. 105 (2), 136–144. 10.1115/1.3138397 6865355

[B14] Gu W. Pandy M. G. (2020). Direct Validation of Human Knee-Joint Contact Mechanics Derived from Subject-specific Finite-Element Models of the Tibiofemoral and Patellofemoral Joints. J. biomechanical Eng. 142 (7), 071001. 10.1115/1.4045594 31802099

[B15] Harris M. D. Cyr A. J. Ali A. A. Fitzpatrick C. K. Rullkoetter P. J. Maletsky L. P. (2016). A Combined Experimental and Computational Approach to Subject-specific Analysis of Knee Joint Laxity. J. biomechanical Eng. 138 (8), 0810041–0810048. 10.1115/1.4033882 PMC4967880 27306137

[B16] Haut Donahue T. L. Hull M. L. Rashid M. M. Jacobs C. R. (2003). How the Stiffness of Meniscal Attachments and Meniscal Material Properties Affect Tibio-Femoral Contact Pressure Computed Using a Validated Finite Element Model of the Human Knee Joint. J. biomechanics 36 (1), 19–34. 10.1016/S0021-9290(02)00305-6 12485635

[B17] Li G. Lopez O. Rubash H. (2001). Variability of a Three-Dimensional Finite Element Model Constructed Using Magnetic Resonance Images of a Knee for Joint Contact Stress Analysis. J. biomechanical Eng. 123 (4), 341–346. 10.1115/1.1385841 11563759

[B18] Maas S. A. Ellis B. J. Ateshian G. A. Weiss J. A. (2012). FEBio: Finite Elements for Biomechanics. J. biomechanical Eng. 134 (1), 011005. 10.1115/1.4005694 PMC3705975 22482660

[B19] Pal S. Besier T. F. Gold G. E. Fredericson M. Delp S. L. Beaupre G. S. (2019). Patellofemoral Cartilage Stresses Are Most Sensitive to Variations in Vastus Medialis Muscle Forces. Comput. Methods Biomechanics Biomed. Eng. 22 (2), 206–216. 10.1080/10255842.2018.1544629 PMC6468979 30596523

[B20] Rooks N. B. Schneider M. T. Y. Erdemir A. Halloran J. P. Laz P. J. Shelburne K. B. (2021). Deciphering the “Art” in Modeling and Simulation of the Knee Joint: Variations in Model Development. J. Biomechanical Eng. 143 (6), 061002. 10.1115/1.4050028 PMC8086182 33537727

[B21] Song Y. Debski R. E. Musahl V. Thomas M. Woo S. L.-Y. (2004). A Three-Dimensional Finite Element Model of the Human Anterior Cruciate Ligament: a Computational Analysis with Experimental Validation. J. biomechanics 37 (3), 383–390. 10.1016/S0021-9290(03)00261-6 14757458

[B22] Zhang J. Fernandez J. Hislop-Jambrich J. Besier T. F. (2016a). Lower Limb Estimation from Sparse Landmarks Using an Articulated Shape Model. J. biomechanics 49 (16), 3875–3881. 10.1016/j.jbiomech.2016.10.021 28573974

[B23] Zhang J. Hislop-Jambrich J. Besier T. F. (2016b). Predictive Statistical Models of Baseline Variations in 3-D Femoral Cortex Morphology. Med. Eng. Phys. 38 (5), 450–457. 10.1016/j.medengphy.2016.02.003 26972387

